# Temporal associations between experiential avoidance and disordered eating behaviors in adolescents and young adults: findings from an epidemiological cohort study with ecological momentary assessment

**DOI:** 10.1007/s40519-023-01584-x

**Published:** 2023-07-05

**Authors:** Stephanie K. V. Peschel, Sophia Fürtjes, Catharina Voss, Christine Sigrist, Johanna Berwanger, Theresa M. Ollmann, Hanna Kische, Frank Rückert, Julian Koenig, Katja Beesdo-Baum

**Affiliations:** 1 Behavioral Epidemiology, Institute of Clinical Psychology and Psychotherapy, Technische Universität Dresden, Dresden, Germany; 2 Department of Child and Adolescent Psychiatry, Psychosomatics and Psychotherapy, Faculty of Medicine and University Hospital Cologne, University of Cologne, Cologne, Germany

**Keywords:** Experiential avoidance, Disordered eating behaviors, Adolescents, Young adults, Ecological momentary assessment, Epidemiology

## Abstract

**Purpose:**

Previous studies linking experiential avoidance (EA) and eating pathology have largely relied on single measurements based on traditional retrospective questionnaires. Taking advantage of available repeated assessments of EA and disordered eating behaviors (DEBs) in the everyday lives of young people from an epidemiological cohort, we aimed to investigate ecologically valid temporal associations between these constructs.

**Methods:**

A random population sample of N = 1180 14–21-year-olds from Dresden, Germany, participated at baseline (2015/2016). As part of smartphone-based ecological momentary assessment (EMA), participants reported on engagement in EA and four DEBs (skipping eating, eating large amounts of food, loss-of-control eating, and restrained eating) up to eight times per day for four days. Multilevel modeling of concurrent and time-lagged associations between EA and DEBs, was conducted among those with at least 50% EMA-compliance (n = 1069).

**Results:**

EA was associated with higher concurrent levels of all four types of concurrent DEBs. In addition, EA significantly predicted subsequent levels of restrained eating. Only loss-of-control eating significantly predicted subsequent EA, and this effect depended on the timespan between consecutive assessments. When this timespan was short, higher Loss-of-control eating predicted lower subsequent EA, while it predicted higher subsequent EA when the timespan was longer.

**Conclusion:**

The present findings suggest that EA is temporally closely linked to greater engagement in DEBs, supporting theoretical assumptions that DEBs may serve an attempted avoidance function in the context of unpleasant inner experiences. Future studies may benefit from examining samples with more pronounced eating pathology.

**Level of evidence:**

Level IV: Evidence obtained from multiple time series with or without the intervention, such as case studies.

**Supplementary Information:**

The online version contains supplementary material available at 10.1007/s40519-023-01584-x.

## Introduction

Approximately every fifth child or adolescent aged between 6 and 18 years experiences problematic eating disorder symptoms [1]. Similarly, specific behavioral eating disorder symptoms, i.e., disordered eating behaviors (DEBs), such as binge eating or restrained eating, are highly prevalent among adolescents and young adults [2–4]. DEBs have been identified as prospective risk factors for the development of clinical eating disorders [5, 6] and are cross-sectionally and prospectively linked to various adverse outcomes, such as suicidality and symptoms of depression [7, 8], psychological distress and impaired quality of life [9], and poor self-rated health [10]. Thus, improving knowledge on processes affecting engagement in DEBs in young people is pivotal to inform prevention and early intervention strategies. One such process that has been discussed as relevant to the engagement in DEBs is experiential avoidance (EA). EA has been conceptualized as the unwillingness to stay in touch with one’s unpleasant inner experiences, such as thoughts, feelings, and physical sensations, resulting in avoidance or other efforts to control these unwanted experiences [11]. Engaging in EA has been associated with increases [12] and overall higher levels of negative affect [13, 14], as well as an increase of unwanted thoughts [11, 15], making it a seemingly ineffective attempt to cope with adverse internal experiences. Consistent with its seemingly maladaptive nature, EA has been connected to a broad spectrum of psychopathology [11, 15], including eating pathology [16]. More specifically, the *Functional model of emotion avoidance in Anorexia Nervosa *[17] and the *Transactional model* *of* *emotion dysregulation in Anorexia Nervosa* [18] suggest that typical DEBs, such as restriction, serve avoidance and escape-functions from negative affect. Similarly, the *Escape theory* suggests that binge eating is aimed at diverting awareness from distressing inner experiences [19].

The significance of EA in eating pathology is corroborated by empirical findings. On the one hand, previous studies have reported higher levels of EA in individuals with clinical eating disorders, including Anorexia Nervosa [17, 20, 21] and Bulimia Nervosa [21], compared to controls. On the other hand, greater EA has also been associated with dimensional eating pathology in non-clinical samples. Two studies have reported associations between higher overall self-reported symptoms of Bulimia Nervosa measured via questionnaire and greater EA [22] as well as related aspects such as thought suppression [23] in university students respectively. Similarly, more pronounced thought suppression has been associated with more severe general eating disorder symptoms in adolescents and young adults [24] measured via the Eating Disorder Examination Questionnaire [25]. Of note, measurements of eating pathology in these three studies solely included single dimensional severity scores of eating disorders symptoms, rather than differentiating between different types of DEBs. In a more specific manner, another study has reported a positive association between greater EA and more severe binge eating pathology in undergraduate students [26]. In addition to often not differentiating between specific kinds of DEBs, previous associations between EA and eating pathology have largely been investigated using cross-sectional analyses based on traditional self-report questionnaires. While these have revealed general links between EA and disordered eating, it is unclear whether and how these two phenomena are related at the situational level in everyday life. On the one hand, given that DEBs, such as restrictive eating or binge eating, have been conceptualized as avoidance behaviors themselves within theoretical models [17–19], it may be assumed that EA is related to concurrent DEBs, with DEBs representing a specific marker of EA. On the other hand, it could also be hypothesized that practicing EA may potentially increase the likelihood of engaging in subsequent DEBs, when the attempt to cope with negative experiences through avoidance has failed. In support of this, some previous studies have suggested that momentary maladaptive emotion regulation through ineffective strategies (e.g., rumination) increases the risk for subsequent binge eating on a situational [27] and on a day-to-day [28] level. With particular relevance to the present work, there is initial evidence that momentary engagement in EA may subsequently increase the type of negative affect that one has been trying to avoid [12]. Negative affect may also promote engagement in DEBs [29, 30], although related empirical evidence has been mixed in adolescents [e.g., 31–33]. Accordingly, one may speculate that EA may increase subsequent DEB-severity through a rebound in negative affect that is potentially dealt with in a dysfunctional manner.

Furthermore, although theoretical models focus on the impact of EA on DEBs [17–19], reverse relations, meaning a potential impact of DEBs on subsequent levels of EA, may also appear conceivable. Restricting food intake [34] and binge eating [30] have been shown to lead to increased negative affect, implying that DEBs may create unpleasant inner experiences, to which individuals may also respond with increased EA.

Elucidating potential temporal associations may further clarify the role of EA in the context of DEBs and may have important implications for treatment or prevention.

To the best of our knowledge, no previous study has examined temporal associations between EA and DEBs in the daily lives of adolescents and young adults. To address this issue, the present study builds on available data from ecological momentary assessment (EMA), i.e., repeated measurements of experiences and behaviors in everyday life and in the natural environment [35], collected as part of a large epidemiological cohort study [36].

Based on the considerations outlined above, we hypothesized that (1) higher EA would be associated with higher concurrent levels of DEBs within the same assessment period. We further hypothesized that (2) higher EA reported within one assessment period would predict higher subsequent levels of DEBs within the subsequent assessment period. As an additional exploratory research question, considering deliberations regarding how DEBs may affect subsequent EA outlined above, we also aimed to investigate whether more severe DEBs within one assessment period would predict subsequent levels of EA within the subsequent assessment period.

## Methods and materials

### Participants and procedure

The present analysis used baseline data from the Behavior and Mind Health (BeMIND) study [36]. The BeMIND study is an epidemiological cohort study program aiming at investigating a large variety of aspects that may influence mental health in adolescents and young adults from the general population. For the first baseline wave, an age- and sex-stratified random sample of 14- to 21-year-olds was drawn from the population registry of the city of Dresden, Germany, in 2015 and a total of N = 1180 (participation/response proportion: 21.7%) were assessed between 11/2015 and 12/2016. Individuals were excluded if they were institutionalized, had insufficient German language skills, or lived outside of the city of Dresden during the field phase. Written informed consent/assent was obtained from all study participants, as well as from legal guardians in the case of underage individuals. Ethical approval for the study was granted by the Ethics Committee of TU Dresden (No. EK381102014) and all study components were conducted in accordance with the Declaration of Helsinki.

The BeMIND baseline investigation included several study components, including two in-person laboratory sessions (approximately 7 days apart) with a clinical diagnostic interview, questionnaires, biosampling, and experimental assessments. Between the two appointments, participants completed online questionnaires and a four-day EMA (see details below). Further detailed information on sampling, study components, and baseline characteristics of the study cohort can be found in Beesdo-Baum et al. [36].

### Ecological momentary assessment

EMA covering various aspects of experiences and behaviors in daily life [36] was completed over 4 consecutive days, including 2 week- and 2 weekend days and was implemented via a self-developed app installed on study smartphones. Participants received a study smartphone upon completion of the first lab-appointment. Study personnel provided a technical introduction as well as a tutorial on how to fill out the questionnaires. EMA-assessments were prompted via an acoustic signal eight times a day (once in the morning, once shortly before going to sleep, six times during the day) under the consideration of individually set wake-, sleep-, and “do-not-disturb”-times. EMA-assessments could be postponed for a maximum of three times (five minutes each) or omitted completely. The minimum time between two consecutive EMA-assessments was 30 min, although in very few cases this was shorter (e.g., if the previous assessment had been postponed). Assessments occurring within less than 30 min after the previous one (n = 20) were removed from the analyses.

To allow for familiarization with the EMA-procedure and a reduction of reactivity, individuals completed up to three EMA practice assessments on the day prior to the four-day EMA period. Practice data are not included in the present analysis. Study smartphones were returned to study personnel at the second in-person appointment, after which data was transferred to the server.

### Measures

#### Experiential avoidance

Levels of EA within the time period since the last assessment was measured at each EMA-assessment using three items adapted from another EMA-study [12]. Items were phrased as statements about the self-reported frequency of experiences and ways of dealing with thoughts and feelings since the last EMA-assessment (see Supplementary S1 for details on all EMA-items used in the present study). Items were rated on a slider scale (0 = never, 50 = sometimes, 100 = always) and evaluated via the mean score, with higher values indicating higher levels of EA.

The three items measuring EA roughly covered urges to both control and avoid one’s feelings and thoughts, as well as intense negative appraisal of one’s feelings and thoughts. These facets are also included in commonly used trait measures of EA, although EA is a multifaceted construct and is typically measured using more than three items [37, 38]. Due to the need to limit participant burden in the present study, EA was covered by a brief measure. To examine validity, based on available data in the analysis sample, Spearman rank correlations between average EA across the assessment period, and two trait questionnaires were inspected: 1) an adapted and abbreviated version of the cognitive avoidance subscale (4 items, available from n = 1010 participants, Cronbach’s alpha = 0.74) from the Coping Responses Inventory [39] as well as the emotion acceptance subscale (3 items, available from n = 971 participants, Cronbach’s alpha = 0.69) from the Emotion-Regulation Skills Questionnaire [40]. In support of the validity of the EA-measure used in the present study, higher EA was positively (albeit weakly) correlated with cognitive avoidance (*ρ* = 0.19, *p* < 0.001) and negatively correlated with emotion acceptance (*ρ* = − 0.23, *p* < 0.001). Multilevel reliabilities (ω) [41] were calculated in Rstudio using the multilevelTools-package (version 0.1.1) [42]. Within-person ω was acceptable (0.72) and between-person ω was excellent (0.93).

Previous analyses from the BeMIND-study involving EA have been published before [43–45], but were unrelated to eating behavior.

#### Disordered eating behaviors

Items measuring levels of DEBs within the time period since the last assessment were also adapted from another EMA-study [46]. In this study [46], items had been adapted from the Eating Disorders Examination Questionnaire [25], thus covering DEBs that are both typical and commonly included in dimensional questionnaires of eating pathology. Participants reported on levels of engagement in DEBs since the last EMA following a filter question, asking them if they had eaten since the last assessment. If they had not, skipping eating to control one’s weight or body shape (hereafter referred to as skipping eating) was assessed. If eating was affirmed, eating large amounts of food, loss-of control eating (LOC) and restrained eating were assessed. Each DEB was assessed with a single item. Items were rated on a continuous slider scale ranging from 0 (not at all)–100 (absolutely), with higher scores reflecting higher levels of DEBs. Generalized linear models demonstrated that individuals with a 12-month eating disorder diagnosis showed consistently higher average levels in all four DEBs across the assessment period (all *p*’s < 0.001), supporting validity of the DEB-measurements in the present study.

#### Mood and negative thoughts

Because negative mood and negative thoughts were assumed to likely co-occur with EA and may also have a direct impact on DEBs, respective measures of levels within the time period since the last assessment were included in the analyses. Within each EMA-assessment, a continuous slider scale was used to rate the quality of mood (0 = negative, 50 = neutral, 100 = positive) and frequency of negative thoughts (0 = no negative thoughts, 100 = very frequent negative thoughts) since the last assessment. Both constructs were measured with one single item each, developed by the BeMIND-study group.

#### Mental disorders

As diagnosed eating disorders are expected to influence levels of DEBs and may also be associated with higher EA [17, 20, 21], the presence of a respective 12-month-diagnosis was considered in the present study. Lifetime and 12-month diagnoses of DSM-5 mental disorders, including eating disorders, were obtained via an updated version (DIA-X-5/D-CIDI) [47] of the standardized and computerized Munich Composite International Diagnostic Interview [48], administered face-to-face by trained interviewers during the first laboratory visit. Eating disorder diagnoses covered by the DIA-X-5 include (atypical) Anorexia Nervosa, Bulimia Nervosa (including of limited duration), binge eating disorder (including of limited duration), purging disorder, and unspecified eating disorders. Conservatively, eating disorder-diagnoses were coded as not fulfilled when respective data were missing (i.e., n = 10 participants did not complete the eating disorder section).

### Analysis sample

Participants were excluded from the present analyses if they did not participate in EMA (n = 26), completed less than 50% of the EMA-assessments yielding insufficient and unreliable data (n = 82), or if EMA-responses were implausible, i.e., participants indicated no eating episode across the entire assessment period (n = 3). This resulted in a final analysis sample of N = 1069 (90.6% of the total sample).

### Statistical analyses

Main statistical analyses were performed using Stata 17 [49]. Additionally, conditional R^2^-approximations (R^2^c; providing an approximate for the variance explained by an entire model) were calculated in Rstudio version 2023.3.0.386 [50] (R version 4.3.0) using the lme4 (version 1.1–33) [51] and MuMIn (version 1.47.5) [52] packages.

Due to the nested structure of the data, we performed multilevel mixed-effects generalized linear models (MEGLMs) with random intercepts. To account for the skewed distribution of DEBs and EA, all models assumed a gamma-distribution and a log link.

Model 1 (relating to hypothesis 1) tested situational EA as a predictor of concurrent situational DEBs (multiple situational observations [level 1] nested within participants [level 2]). Hypothesis 2 was tested in a similar manner, except that EA as a predictor was time-lagged. More specifically, Model 2 tested whether situational EA at *t-1* would predict subsequent situational DEBs at *t*.

Finally, in an exploratory fashion, Model 3 examined whether situational DEBs at *t-1* would predict subsequent situational EA at *t.*

In models including time-lagged predictors, only assessments less than three hours apart from each other were considered consecutive.

Each model was run separately for each DEB, representing either the dependent variable (Model 1 and 2), or the independent variable (Model 3). Since models assuming a gamma-distribution cannot accommodate values of zero in dependent variables, a constant of 1 added to each individual DEB-value (Model 1 and Model 2) and to each individual EA-scores (Model 3).

All models were adjusted for level 1 scores of mood and negative thoughts, sex, age, the presence/absence of a 12-month eating disorder diagnosis, and the BMI-standard deviation score (BMI-SDS; refers to an individual’s standard deviation from the age-and sex specific BMI-median based on national norms [53–55]). Model 1 and Model 2 were further adjusted for each individual’s total average EA across the EMA-period, while Model 3 was adjusted for the respective total average DEB-level across the EMA-period (e.g., when level 1 restrained eating represented the predictor, the model was adjusted or each individual’s average level in restrained eating).

Model 2 and Model 3 also included the time difference between two consecutive assessments and the interaction between the lagged situational predictors (EA and DEBs respectively) and the time difference as covariates to account for varying time periods between assessments.

Level 1 predictors at the situational level (except time difference) were person-mean centered to reflect within-person fluctuations.

Analyses were weighted to increase representativeness in terms of age and sex with respect to 14–21-year-olds living in Dresden. Based on all possible combinations of age and sex, the sample was split into 16 strata. Sample weights were calculated and applied so that the strata in the present sample would correspond to the respective strata of 14–21-year-olds in Dresden. In brief, sample weights were applied so that female participants would receive a lower weight, and older individuals would receive a higher weight, to better resemble the target population. For a descriptive illustration, weighted and unweighted descriptives of age and sex are provided in Table 1. For more detailed information, please refer to a previous publication [36].

**Table 1:** Sample characteristics and descriptive values of the main study variables

Variables	Included participants n = 1069
Sex, n (%, *w*%) female	629 (58.84, 48.86)
Age, mean (SD), *w*mean (*w*SD)	17.27 (2.28), 17.95 (2.34)
BMI-SDS, *w*mean (SD)	0.10 (0.94)
German nationality, n (*w*%)	1043 (97.21)
Education, n (*w*%)	
Low	17 (1.68)
Middle	203 (17.65)
High	815 (78.18)
Other	34 (2.49)
Severities of DEBs	
Skipping eating, *w*mean^a^ (*w*SD)	5.34 (11.26)
Eating large amounts of food, *w*mean (*w*SD)	5.32 (8.75)
Loss-of-control eating, *w*mean (*w*SD)	5.11 (9.72)
Restrained eating, *w*mean (*w*SD)	10.34 (16.95)
Experiential avoidance, *w*mean (*w*SD)	5.01 (7.64)
Mood, *w*mean (*w*SD)	65.15 (12.54)
Negative thoughts, *w*mean (*w*SD)	20.07 (13.62)
12-month-ED diagnosis n (*w*%)	62 (5.18)
EMA-compliance in %, mean (SD)	84.69 (12.90)

*BMI-SDS* body mass index standard deviations score, *ED* eating disorder, *EMA* ecological momentary assessment, *SD* standard deviation, *w* weighted (weights were applied to increase representativeness regarding sex and age, frequencies (n's) and compliance are consistently reported unweighted)

^a^Available from n = 1068

Given the overall exploratory nature of the present analyses, we refrained from adjusting for multiple testing [56]. All analyses were performed with available data and without imputation. Further information on the inclusion/exclusion of participants and EMA-assessments in the statistical models is provided in Supplement S2.

## Results

### Descriptive statistics

Demographic information and descriptive values of the main study variables are provided in Table 1. Information on group differences between the analysis sample and excluded participants is provided in Supplements S3 and S4.

In Model 1, the numbers of included assessments were: skipping eating n = 15,212, LOC n = 13,357; eating large amounts of food n = 13,357; restrained eating n = 13,357.

In Model 2, the numbers of included assessments were: skipping eating n = 11,203; LOC n = 10,017; eating large amounts of food n = 10,018; restrained eating n = 10,017.

In Model 3, the numbers of included assessments were: skipping eating n = 11,280; LOC n = 9946; eating large amounts of food n = 9947; restrained eating n = 9946.

### Concurrent associations

For reasons of clarity, only statistics for the main study variables relevant to our hypotheses are presented here. For tables displaying complete statistics including adjustment variables and R^2^c for all models, please see Supplementary S5–S7.

**Fig. 1:**
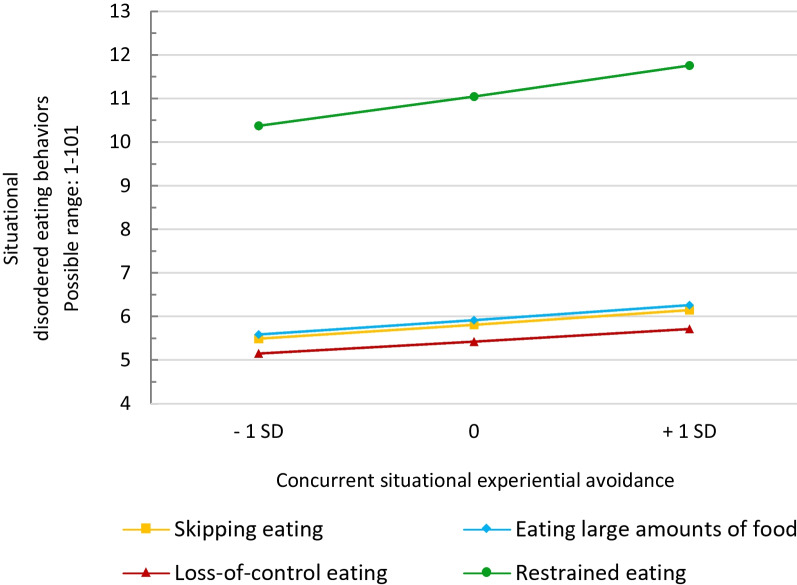
Predictive margins of situational disordered eating behaviors based concurrent levels of situational experiential avoidance (Model 1). Experiential avoidance is person-mean centered. *SD* standard deviation

On the situational level (Model 1), EA was significantly associated with higher levels of concurrent skipping eating (b = 0.007, CI95% 0.004–0.011, *p* < 0.001, R^2^c = 0.401), eating large amounts of food (b = 0.007, CI95% 0.003–0.012, *p* = 0.001, R^2^c = 0.314), LOC (b = 0.007, CI95% 0.002–0.011, *p* = 0.002, R^2^c = 0.334), and restrained eating (b = 0.008, CI95% 0.004–0.013, *p* < 0.001, R^2^c = 0.447). Predictive margins of Model 1 for each DEB are shown in Fig. 1.

### Time-lagged associations between EA and subsequent DEBs

**Fig. 2:**
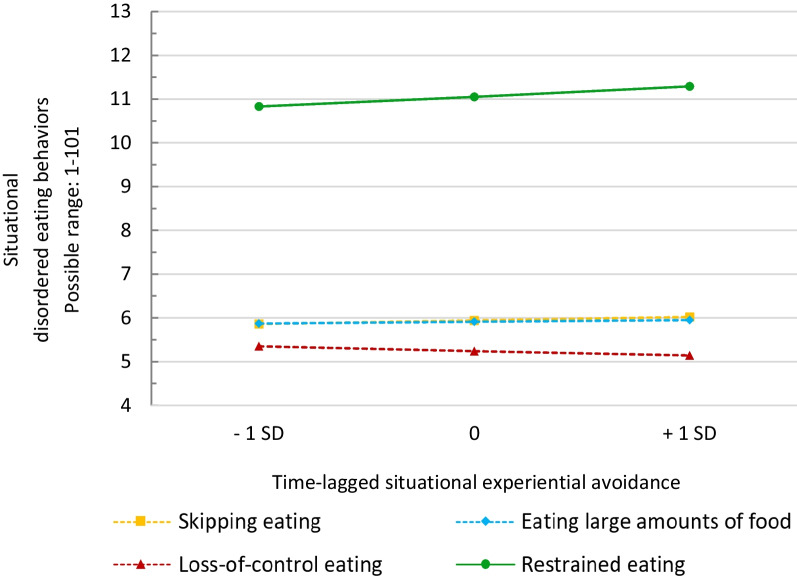
Predictive margins of levels of situational disordered eating behaviors based on time-lagged situational experiential avoidance (Model 2). Experiential avoidance is person-mean centered. Dotted lines indicate non-significant associations. *SD* standard deviation

On the situational level (Model 2), EA significantly predicted subsequent levels of restrained eating (b = 0.032, CI95% 0.001–0.064, *p* = 0.045, R^2^c = 0.468). However, EA did not predict subsequent skipping eating, eating large amounts of food, or LOC (all *p*’s > 0.10). Predictive margins according to Model 2 are displayed in Fig. 2.

### Exploratory analyses: time-lagged associations between DEBs and subsequent EA

**Fig. 3:**
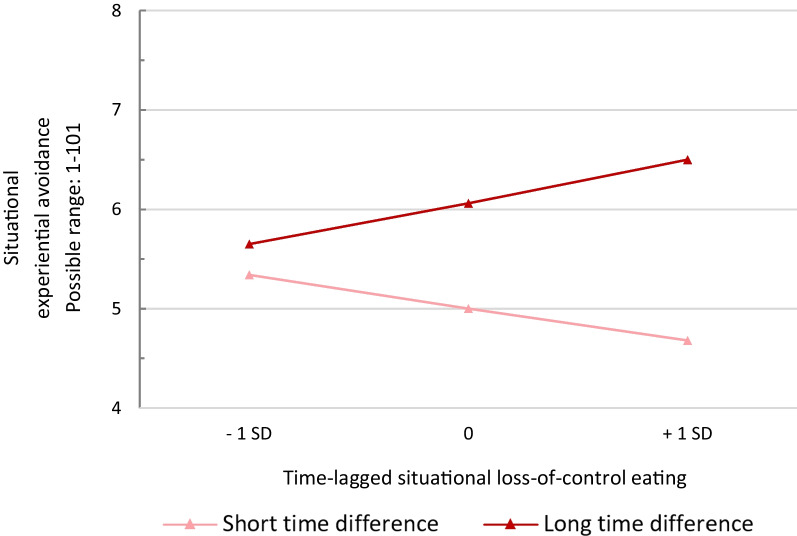
Predictive margins of situational experiential avoidance based on time-lagged situational loss-of-control eating, modulated by the time difference between consecutive assessments. Loss-of-control eating is person-mean centered. *SD* standard deviation

Within Model 3, skipping eating, eating large amounts of food, and restrained eating did not predict subsequent levels of EA (all *p*’s > 0.10). LOC emerged as a significant predictor for subsequent EA (b = − 0.012, CI95% − 0.023 to − 0.001, *p* = 0.033, R^2^c = 0.367). There was also a significant LOC X time difference interaction (b = 0.0001, CI95% 0.000–0.0002, *p* = 0.032), indicating that associations between LOC and subsequent EA were modulated by the timespan between consecutive assessments. Mean time difference between consecutive assessments was 115.50 min (SD = 18.23). Post-hoc analyses revealed that, when this timespan was short, higher levels of LOC predicted lower subsequent levels of EA, whereas higher levels of LOC predicted higher levels of EA when this timespan was long. Predictive margins of EA as predicted by LOC and respective moderation by time difference is displayed in Fig. 3.

## Discussion

The present study investigated temporal associations between EA and four different DEBs in the daily lives of young people from the general population. At the situational level, higher levels of EA relative to one’s mean were associated with higher concurrent levels of all four DEBs, even when adjusting for average EA, mood, negative thoughts, and other relevant covariates. These findings indicate that EA is linked to concurrent DEBs in close temporal proximity. This offers support for the assumption of theoretical models proposing that different DEBs may represent attempts to avoid unpleasant inner experiences [17–19], and is also consistent with previous reports on positive associations between EA and DEBs based on cross-sectional investigations [22–24, 26].

In terms of time-lagged associations, the present study suggests that EA specifically predicts greater restrained eating, but not other types of DEBs, within the next assessment period. Since the present study only examined direct effects of EA on subsequent restrained eating, we can only speculate about potential relevant mechanisms by which this association arises. Considering that EA may increase negative affect [12] and facets of negative affect have also been shown to predict subsequent restrained eating in college students [57], it appears conceivable that greater EA may lead to more severe subsequent restrained eating via an amplification of negative affect. However, these theoretical deliberations require further rigorous investigation.

Further, the present study did not find significant associations between EA and subsequent disinhibited DEBs, which contrasts a previous study reporting that rumination, another maladaptive way of dealing with emotions, predicted subsequent binge eating [27]. Thus, the significance of EA in promoting subsequent engagement in DEBs may differ between types of DEBs.

Moreover, exploratory analyses revealed that more severe LOC predicted lower subsequent EA when the time period between consecutive measurements was short, but predicted higher EA when the time period was longer. The Escape theory [19] suggests that negative affect that may have been temporarily avoided during disinhibited eating will likely return afterwards. In line with the latter assumption, LOC has been previously associated with higher levels of post-meal guilt in children and adolescents [32]. With respect to the present findings, one might speculate that LOC potentially helps to avoid unpleasant inner experiences in the very short-term, requiring less EA right after engaging in disinhibited eating. However, assuming a subsequent increase in negative affect, higher EA may reoccur as a related dysfunctional coping mechanism over the course of time.

Critically, in order to further elucidate bidirectional time-lagged associations between EA and engagement in DEBs and the mechanisms by which the two are related, it appears crucial to examine how momentary EA interacts with unpleasant inner experiences (particularly with affect), and how these interactions may be associated with DEBs in everyday life.

Of note, based on the predictive margins, all significant associations between level-1 EA and DEBs in the current study appeared to be small. This may be due to the overall low severity of DEBs in the general population-derived analysis sample, where low average levels of DEBs were observed and more severe DEBs tended to be rare. While this is to be expected in a non-clinical sample, future EMA-investigations in (sub-)clinical samples are warranted in order to clarify the role of momentary EA and DEBs in individuals with more severe eating pathology. Importantly, it has been recently demonstrated that momentary processes related to binge eating appear to be highly individual [58], which may also apply to relations between EA and different types of DEBs. Since the present study, to the best of our knowledge, was the first one to investigate temporal associations between EA and DEBs, we were primarily interested in the presence or absence of average effects. However, future studies may benefit from investigating random slopes and potential moderators of these relations, such as age, sex or general psychopathology.

Nonetheless, given that the present findings support the role of EA in more pronounced DEBs, targeting EA in respective prevention and intervention approaches including components from Acceptance Commitment Therapy (ACT) [59] seems promising. Given that the present findings support the assumption that DEBs represent an attempt at avoiding unpleasant thoughts and feelings, this may particularly entail promoting acceptance of such experiences as an alternative and more functional way of coping. Previously, ACT-based approaches in the context of eating disorders have put an emphasis on fostering acceptance of distressing thoughts and feelings related to eating and the body (e.g., shape and weight concerns) [e.g., 60, 61]. However, cross-sectional empirical findings indicate that EA may also mediate relationships between symptoms of depression and anxiety and eating pathology [17]. Thus, it may appear beneficial to promote acceptance with respect to a broader range of unpleasant inner processes. While ACT has been shown to reduce eating disorder symptoms in clinical individuals [60, 62], the effectiveness of respective components targeting EA in the context of a broad spectrum of DEBs, including subclinical presentations in young people, should be further examined in the future.

### Strengths and limits

To the best of our knowledge, the present study is the first to investigate ecologically valid temporal associations between EA and DEBs in the daily lives of adolescents and young adults using EMA-data, thus advancing the knowledge on the relationship between the two phenomena. In addition, we investigated a large sample of adolescents and young adults from an epidemiological study that was diverse regarding age, sex, and weight, which allows generalization of the findings to the general population. However, several limitations should be considered. (1) As the BeMIND study was not specifically designed to address the current research question, hypotheses were not pre-registered, and analyses were not adjusted for multiple testing, the present findings must be considered exploratory and require replication in confirmatory studies. (2) The EMA-assessment period of four days was relatively short, which may have resulted in reduced reliability in measuring the processes of interest. The short duration was chosen in the BeMIND study to limit the overall burden of the extensive assessments on the participants. Longer EMA-periods are recommended for future studies. (3) Engagement in DEBs was reported since the last assessment, rather than at a specific point in time. Therefore, the time resolution of present EMA-assessments is limited. In future studies, the inclusion of event-contingent reports of DEBs (i.e., self-initiated assessments whenever participants engage in DEBs) may be particularly beneficial in investigating potential time-lagged effects of EA. (4) With respect to the types of unpleasant experiences, the present study assessed EA only with respect to general thoughts and feelings. In future studies, assessing the interplay between DEBs, EA, and momentary experiences of particular relevance to DEBs that have been previously linked to EA via regular questionnaires, such as body dissatisfaction [24], may further elucidate the specific involvement of EA in eating pathology. (5) While the present study adapted items measuring EA from a previous EMA-study, the validity of the present EA-measure is somewhat unclear, although we were able to demonstrate some results tentatively supporting validity. It has been highlighted that EA is a multifaceted construct [38], including cognitive, affective and/or behavioral components [15]. Accordingly, future studies should develop validated measures for EA within EMA-studies that capture the various facets of EA in a more fine-grained manner, thus also allowing to examine potentially differential associations with DEBs. Finally, (6) the present study investigated a regional sample characterized by predominantly high education, thus potentially limiting generalizability to adolescents and young adults from other regions or countries.

## Conclusions

The results of the present study suggest that higher situational EA is associated with more severe concurrent DEBs in the daily lives of young people. Time-lagged associations between the two phenomena seem less consistent and appear to depend on the type of DEB. Reducing avoidance and fostering more adaptive ways of coping with unpleasant inner experiences may be a promising target for intervention in the context of DEBs. Given the exploratory nature of the present study, confirmatory studies, including investigations in samples with more pronounced eating pathology, are needed.

### What is already known on this subject?

Several theoretical models of disordered eating suggest that disordered eating behaviors are aimed at avoiding negative inner experiences. Furthermore, a number of previous cross-sectional studies have found that experiential avoidance is associated with eating disorder symptoms.

### What does this study add?

Previous studies on associations between experiential avoidance and disordered eating behaviors have largely relied on cross-sectional investigations based on traditional questionnaires. The present work extends related insights by examining temporal associations between the two phenomena in the daily lives of adolescents and young adults via ecological momentary assessment. Our findings suggest that experiential avoidance is closely connected to higher levels of different concurrently-reported disordered eating behaviors and may predict higher subsequent restrained eating. This may imply that disordered eating behaviors serve an avoidance function as proposed by theoretical models. Conversely, loss-of-control eating may also predict subsequent levels of experiential avoidance.

## Supplementary Information

Below is the link to the electronic supplementary material.

Supplementary file1 (DOCX 60 KB)

## Data Availability

The data that support the findings of this study are available from the senior author upon reasonable request.
