# Supplementary material for: Temporal associations between experiential avoidance and disordered eating behaviors in adolescents and young adults: findings from an epidemiological cohort study with ecological momentary assessment
Source: Eat Weight Disord. 2023 Jul 5;28(1):58. doi: 10.1007/s40519-023-01584-x (PMC10322765; doi:10.1007/s40519-023-01584-x)
Supplement: Supplementary file 1 — Supplementary file1 (DOCX 60 KB) [file 40519_2023_1584_MOESM1_ESM.docx]

**Supplementary material**

**Temporal associations between experiential avoidance and disordered eating behaviors in adolescents and young adults: findings from an epidemiological cohort study with ecological momentary assessment**

**Eating and Weight Disorders – Studies on Anorexia, Bulimia and Obesity**

Stephanie K.V. Peschel^1^*, MS, Email: [stephanie.peschel@tu-dresden.de](mailto:stephanie.peschel@tu-dresden.de)

Sophia Fürtjes^1^, PhD

Catharina Voss^1^, MS

Christine Sigrist^2^, PhD

Johanna Berwanger^1^, MS

Theresa M. Ollmann^1^, PhD

Hanna Kische^1^, PhD

Frank Rückert^1^, MS

Julian Koenig^2^, PhD

Katja Beesdo-Baum^1^, PhD, Email: [katja.beesdo-baum@tu-dresden.de](mailto:katja.beesdo-baum@tu-dresden.de)

^1^Behavioral Epidemiology, Institute of Clinical Psychology and Psychotherapy, Technische Universität Dresden, Dresden, Germany

^2^ Department of Child and Adolescent Psychiatry, Psychosomatics and Psychotherapy, Faculty of Medicine and University Hospital Cologne, University of Cologne, Cologne, Germany

*Corresponding author

**S1. Items from ecological momentary assessment in English (translated) and German (original)**

| **Construct** | **English item wording** | **Rating in English** | **German item wording** | **Rating in German** |
| --- | --- | --- | --- | --- |
| **Experiential Avoidance**  (adapted from [1]) | Since the last assessment… I was upset or bothered about my feelings or thoughts. | seekbar  0 = never, 50 = sometimes, 100 = always | Seit der letzten Abfrage… war ich aufgebracht oder beunruhigt über meine Gefühle oder Gedanken. | Seekbar  0 = nie, 50 = manchmal, 100 = immer |
|  | Since the last assessment… I have been trying hard to get rid of my feelings and thoughts. | seekbar  0 = never, 50 = sometimes, 100 = always | Seit der letzten Abfrage… habe ich mich angestrengt, meine Gefühle und Gedanken loszuwerden. | Seekbar  0 = nie, 50 = manchmal, 100 = immer |
|  | Since the last assessment… I gave up saying or doing what I really wanted or mattered to me in order to control or manage my thoughts/feelings. | seekbar  0 = never, 50 = sometimes, 100 = always | Seit der letzten Abfrage… habe ich aufgehört, das zu sagen oder zu tun, was ich eigentlich wollte oder was mir viel bedeutet, um meine Gedanken/Gefühle zu kontrollieren oder zu bewältigen. | Seekbar  0 = nie, 50 = manchmal, 100 = immer |
| **Disordered Eating Behaviors**  (adapted from [2]) | You have stated that you have not eaten since the last assessment. Was one reason not to eat the attempt to control your weight or body shape? | seekbar  0 = not at all, 100 = absolutely | Du hast angegeben, seit der letzten Abfrage nichts gegessen zu haben. War ein Grund dafür nichts zu essen der Versuch Dein Gewicht oder Deine Körperform zu kontrollieren? | Seekbar  0 = überhaupt nicht, 100 = absolut |
|  | Since the last assessment… I ate as if in a rush or I ate a large amount of food given the circumstances | seekbar  0 = not at all, 100 = absolutely | Seit der letzten Abfrage… habe ich wie im Rausch, oder unter den gegebenen Umständen eine große Menge gegessen. | Seekbar  0 = überhaupt nicht, 100 = absolut |
|  | Since the last assessment… I lost control over my eating behavior | seekbar  0 = not at all, 100 = absolutely | Seit der letzten Abfrage… habe ich die Kontrolle über mein Essverhalten verloren. | Seekbar  0 = überhaupt nicht, 100 = absolut |
|  | Since the last assessment… I tried to limit the amount of food that I ate | seekbar  0 = not at all, 100 = absolutely | Seit der letzten Abfrage… habe ich versucht, die Menge der Lebensmittel, die ich gegessen habe, zu begrenzen. | Seekbar  0 = überhaupt nicht, 100 = absolut |
| **Mood** | Since the last assessment, my mood has been… | seekbar  0 = negative, 50 = neutral, 100 = positive | Seit der letzten Abfrage war meine durchschnittliche Stimmung… | Seekbar  0 = negativ, 50 = neutral, 100 = positiv |
| **Negative thoughts** | Since the last assessment… I have thought about negative or unpleasant things. | seekbar  0 = no negative thoughts, 100 = very frequent negative thoughts | Seit der letzten Abfrage… habe ich an negative oder unangenehme Dinge gedacht. | Seekbar  0 = keine negativen Gedanken, 100 = sehr häufig negative Gedanken |

**S2. Further information on inclusion and exclusion of participants and assessments from multilevel models**

This paragraph provides further information on the number of excluded and included assessments/observations as well as participants in the specific multilevel mixed-effects generalized linear models (MEGLMs) that were executed for hypothesis testing.

Three participants had one EMA-assessment each where values were entered both for skipping eating and other disordered eating behaviors, which should not have occurred due to the filter item assessing confirmation/denial of food intake (i.e., individuals reporting on skipping eating should not have been concurrently presented with any of the other disordered eating items within the same EMA-assessment). Presuming technical difficulties, single values for all four disordered eating behaviors were removed from these assessments. Moreover, one participant confirmed food intake at every assessment. Thus, this participant provided no values for skipping eating and was excluded from all models including skipping eating as the outcome.

The minimum time between two consecutive EMA-assessments was 30 minutes, although in very few cases this was shorter (e.g., if the previous assessment had been postponed). Assessments occurring within less than 30 minutes after the previous one (n = 20) were removed from the analyses.

In MEGLMs, only assessments/observations in which values for all predictor variables (including level-1 covariates) were available, were included. Not all participants could be included in time-lagged analyses (Model 2 and Model 3; i.e., if no assessments were available in which experiential avoidance and disordered eating behaviors were measured in the timeframe required for time-lagged analyses). In the following, we provide specific information on the number of included participants and included assessments/observations in the MEGLMs for hypothesis-testing.

*In Model 1*, the numbers of included participants were: skipping eating n = 1,068; LOC n = 1,069; eating large amounts of food n = 1,069; restrained eating n = 1,069. The numbers of included assessments (situational level) were: skipping eating n = 15,212, LOC n = 13,357; eating large amounts of food n = 13,357; restrained eating n = 13,357.

*In Model 2*, the numbers of included participants were: skipping eating n = 1,067; LOC n = 1,065; eating large amounts of food n = 1,065; restrained eating n = 1,065. The numbers of included assessments (situational level) were: skipping eating n = 11,203; LOC n = 10,017; eating large amounts of food n = 10,018; restrained eating n = 10,017.

*In Model 3*, the numbers of included participants were: skipping eating n = 1,065; LOC n = 1,063; eating large amounts of food n = 1,063; restrained eating n = 1,063. The numbers of included observations (situational level) were: skipping eating n = 11,280; LOC n = 9,946; eating large amounts of food n = 9,947; restrained eating n = 9,946.

**S3. Group differences between included and excluded participants**

Descriptive statistics and statistics regarding group differences between included and excluded participants are provided in Table S4. Data as well as tests examining group differences were weighted to increase the representativeness regarding sex and age with respect to 14-21-year-olds living in Dresden. Only frequencies (n’s) and EMA-compliance are reported unweighted.

Group differences in continuous variables (age, BMI-SDS, mean scores of EMA-variables, EMA-compliance) were examined using linear regressions with group (included vs. excluded) as the predictor variable and the included sample being set as the base group.

Group differences in dichotomous variables (sex, nationality, 12-month eating disorder diagnosis) were examined using logistic regressions, again with group (included vs. excluded) as the predictor variable and the included sample being set as the base group.

Group differences in education, i.e., a categorical variable with multiple categories, were first globally examined via a design-based F-test [3] to establish if there were any differences between groups. Given the significant result of the global test, we further investigated pairwise comparisons between the included and excluded group.

| **Table S4.** Sample characteristics and descriptive values of the main study variables including group differences between included and excluded participants. | | | |  |
| --- | --- | --- | --- | --- |
|  |  |  |  |  |
| **Variables** | **Included participants n = 1,069** | **Excluded participants n = 111** | **Group difference included vs. excluded participants** |  |
| Sex, n (*w*%) female | 629 (48.86) | 56 (42.57) | OR = 0.78, 95%CI (0.51, 1.17), *p* = .227 |  |
| Age, mean (SD) | 17.95 (2.34) | 17.72 (2.22) | b = -0.23, 95%CI (-0.70, 0.24), *p* = .340 |  |
| BMI-SDS, mean (SD) | 0.10 (0.94) | 0.08 (1.03)^+^ | b = -0.01, 95%CI (-0.22, 0.19), *p* = .897 |  |
| German nationality, n (*w*%) | 1043 (97.21) | 107 (96.32) | OR = 1.33, 95%CI (0.44, 4.00), *p* = .610 |  |
| Education, n (*w*%) |  |  | design‐based F(2.92, 3444.59) = 9.03 , ***p* < .001** |  |
| low | 17 (1.68) | 8 (8.22) | design‐based F(1,1179) = 14.58, ***p* < .001** |  |
| middle | 203 (17.65) | 29 (26.36) | design‐based F(1, 1179) = 4.35 , ***p* = .037** |  |
| high | 815 (78.18) | 67 (60.03) | design‐based F(1, 1179) = 16.07 , ***p* < .001** |  |
| other | 34 (2.49) | 7 (5.39) | design‐based F(1, 1179) = 3.59 , *p* = .058 |  |
| Severities of DEBs |  |  |  |  |
| Skipping eating, mean (SD) | 5.34 (11.26)^§^ | 8.14 (14.25)^++^ | b = 2.81, 95%CI (-0.79, 6.40), *p* = .126 |  |
| Eating large amounts of food, mean (SD) | 5.32 (8.75) | 9.51 (12.23)^+++^ | b = 4.19, 95%CI (1.38, 6.99), ***p* = .003** |  |
| Loss-of-control eating, mean (SD) | 5.11 (9.72) | 7.82 (13.58)^+++^ | b = 2.70, 95%CI (-0.39, 5.79), *p* = .086 |  |
| Restrained eating,  mean (SD) | 10.34 (16.95) | 14.99 (21.35)^+++^ | b = 4.65, 95%CI (-0.35, 9.65), *p* = .068 |  |
| Experiential avoidance, mean (SD) | 5.01 (7.64) | 9.14 (11.05)^#^ | b = 4.13, 95%CI (1.65, 6.60), ***p* = .001** |  |
| Mood, mean (SD) | 65.15 (12.54) | 63.36 (14.67)^##^ | b = -1.79, 95%CI (-5.07, 1.49), *p* = .284 |  |
| Negative thoughts, mean (SD) | 20.07 (13.62) | 23.07 (15.28)^##^ | b = 3.01, 95%CI (-0.67, 6.68), *p* = .109 |  |
| 12-month-ED diagnosis n (*w*%) | 62 (5.18) | 5 (4.33) | OR = 0.83, 95%CI (0.31, 2.19), *p* = .704 |  |
| EMA-compliance in %, mean (SD) | 84.50 (12.92) | 33.35 (14.61)^##^ | b = -51.16, 95%CI (-54.04, -48.27), ***p* < .001** |  |
| *Note.* Data are weighted to increase representativeness regarding sex and age, frequencies (n's) and compliance are reported unweighted. Abbreviations: BMI-SDS, body mass index standard deviations score; ED, eating disorder; EMA, ecological momentary assessment; SD, standard deviation. *p*-values < .05 are printed in bold.  ^§^ available from n = 1,068  ^+^ available from n = 105  ^++^ available from n = 82  ^+++^ available from n = 80  ^#^ available from n = 84  ^##^ available from n = 85 | | | |  |
|  |  |  |  |  |
|  |  |  |  |  |

| **Table S5.** Associations between experiential avoidance and concurrent disordered eating behaviors on the situational level (Model 1) | | | | | | | | | | | | | | | | |  |
| --- | --- | --- | --- | --- | --- | --- | --- | --- | --- | --- | --- | --- | --- | --- | --- | --- | --- |
|  |  |  |  |  |  |  |  |  |  |  |  |  |  |  |  |  |  |
|  | **Skipping eating** | | | | **Eating large amounts of food** | | | | **Loss-of-control eating** | | | | **Restrained eating** | | | |  |
| Fixed effects | b | SE | 95% CI | *p* | b | SE | 95% CI | *p* | b | SE | 95% CI | *p* | b | SE | 95% CI | *p* |  |
| Intercept | 0.837 | 0.223 | 0.400 - 1.274 | **<.001** | 0.913 | 0.232 | 0.459 - 1.368 | **<.001** | 1.140 | 0.230 | 0.690 - 1.591 | **<.001** | 1.653 | 0.287 | 1.089 - 2.216 | **<.001** |  |
| Momentary experiential avoidance | 0.007 | 0.002 | 0.004 - 0.011 | **<.001** | 0.007 | 0.002 | 0.003 - 0.012 | **.001** | 0.007 | 0.002 | 0.002 - 0.011 | **.002** | 0.008 | 0.002 | 0.004 - 0.013 | **<.001** |  |
| Average experiential avoidance | 0.051 | 0.005 | 0.041 - 0.060 | **<.001** | 0.049 | 0.005 | 0.038 - 0.060 | **<.001** | 0.047 | 0.005 | 0.037 - 0.057 | **<.001** | 0.043 | 0.005 | 0.033 - 0.054 | **<.001** |  |
| Momentary mood | 0.002 | 0.001 | 0.000 - 0.003 | **.018** | 0.003 | 0.001 | 0.001 - 0.004 | **.005** | 0.001 | 0.001 | -0.001 - 0.003 | .221 | 0.001 | 0.001 | -0.001 - 0.003 | .361 |  |
| Momentary negative thoughts | 0.002 | 0.001 | 0.001 - 0.003 | **.005** | 0.002 | 0.001 | -0.000 - 0.003 | .058 | 0.002 | 0.001 | 0.001 - 0.004 | **.005** | 0.001 | 0.001 | -0.000 - 0.003 | .086 |  |
| Sex ^a^ | 0.030 | 0.062 | -0.092 - 0.151 | .633 | - 0.056 | 0.060 | -0.174 - 0.061 | .347 | 0.053 | 0.057 | -0.059 - 0.166 | .355 | 0.130 | 0.075 | -0.017 - 0.277 | .083 |  |
| Age | -0.000 | 0.013 | -0.025 - 0.024 | .980 | 0.008 | 0.013 | -0.017 - 0.034 | .525 | -0.012 | 0.013 | -0.037 - 0.013 | .361 | -0.022 | 0.016 | -0.053 - 0.010 | .175 |  |
| ED-diagnosis ^b^ | 0.759 | 0.222 | 0.324 - 1.195 | **.001** | 0.323 | 0.156 | 0.018 - 0.628 | **.038** | 0.410 | 0.163 | 0.090 - 0.730 | **.012** | 0.980 | 0.219 | 0.550 - 1.410 | **<.001** |  |
| BMI-SDS | 0.156 | 0.035 | 0.087 - 0.225 | **<.001** | 0.090 | 0.033 | 0.026 - 0.154 | **.006** | 0.130 | 0.032 | 0.068 - 0.192 | **<.001** | 0.293 | 0.041 | 0.213 - 0.374 | **<.001** |  |
| Random effects | variance | SE | 95% CI |  | variance | SE | 95% CI |  | variance | SE | 95% CI |  | variance | SE | 95% CI |  |  |
| Intercept | 0.785 | 0.044 | 0.703 - 0.877 |  | 0.753 | 0.037 | 0.684 - 0.829 |  | 0.730 | 0.041 | 0.654 - 0.814 |  | 1.193 | 0.054 | 1.091 - 1.304 |  |  |
| R^2^c | 0.401 | | | | 0.314 | | | | 0.334 | | | | 0.447 | | | |  |

*Note.* Abbreviations: ED, eating disorder; R^2^c, conditional R^2^-approximation; SE, standard errors (robust standard errors are displayed). Analyses are weighted with respect to age and sex. *p*-values < .05 are printed in bold. Experiential avoidance, mood, and negative thoughts are person-mean-centered.

^a^ male sex represents the base category.

^b^ absence of a 12-month eating disorder diagnosis represents the base category.

| **Table S6.** Associations between time-lagged experiential avoidance and subsequent disordered eating behaviors on the situational level (Model 2) | | | | | | | | | | | | | | | | |  |
| --- | --- | --- | --- | --- | --- | --- | --- | --- | --- | --- | --- | --- | --- | --- | --- | --- | --- |
|  |  |  |  |  |  |  |  |  |  |  |  |  |  |  |  |  |  |
|  | **Skipping eating** | | | | **Eating large amounts of food** | | | | **Loss-of-control eating** | | | | **Restrained eating** | | | |  |
| Fixed effects | b | SE | 95% CI | *p* | b | SE | 95% CI | *p* | b | SE | 95% CI | *p* | b | SE | 95% CI | *p* |  |
| Intercept | 0.800 | 0.246 | 0.318 - 1.282 | **.001** | 0.875 | 0.257 | 0.371 - 1.379 | **.001** | 1.029 | 0.243 | 0.554 - 1.505 | **< .001** | 1.547 | 0.306 | 0.946 - 2.147 | **< .001** |  |
| Time-lagged experiential avoidance | 0.016 | 0.012 | -0.007 - 0.039 | .180 | 0.013 | 0.012 | -0.011 - 0.037 | .282 | 0.018 | 0.013 | -0.008 - 0.043 | .179 | 0.032 | 0.016 | 0.001 - 0.064 | **.045** |  |
| Time difference | 0.001 | 0.001 | -0.001 - 0.002 | .427 | -0.000 | 0.001 | -0.002 - 0.001 | .744 | 0.000 | 0.001 | -0.002 - 0.002 | .979 | 0.001 | 0.001 | -0.001 - 0.003 | .263 |  |
| Time difference X Time-lagged experiential avoidance | -0.000 | 0.000 | -0.000 - 0.000 | .231 | -0.000 | 0.000 | -0.000 - 0.000 | .303 | -0.000 | 0.000 | -0.000 - 0.000 | .111 | -0.000 | 0.000 | -0.001 - 0.000 | .076 |  |
| Average experiential avoidance | 0.051 | 0.005 | 0.041 - 0.062 | **< .001** | 0.049 | 0.006 | 0.038 - 0.060 | **< .001** | 0.048 | 0.005 | 0.037 - 0.058 | **< .001** | 0.044 | 0.006 | 0.033 - 0.055 | **< .001** |  |
| Time-lagged mood | 0.001 | 0.001 | -0.001 - 0.002 | .400 | -0.000 | 0.001 | -0.003 - 0.002 | .644 | -0.001 | 0.001 | -0.004 - 0.001 | .145 | 0.003 | 0.001 | 0.001 - 0.005 | **.012** |  |
| Time-lagged negative thoughts | 0.001 | 0.001 | -0.001 - 0.002 | .361 | -0.001 | 0.001 | -0.003 - 0.001 | .299 | 0.001 | 0.001 | -0.001 - 0.003 | .310 | 0.001 | 0.001 | -0.000 - 0.003 | .145 |  |
| Sex ^a^ | 0.035 | 0.064 | -0.091 - 0.161 | .590 | -0.067 | 0.063 | -0.190 - 0.056 | .288 | 0.040 | 0.059 | -0.077 - 0.156 | .504 | 0.116 | 0.077 | -0.034 - 0.267 | .131 |  |
| Age | -0.002 | 0.013 | -0.028 - 0.023 | .848 | 0.012 | 0.014 | -0.015 - 0.039 | .381 | -0.008 | 0.013 | -0.033 - 0.018 | .555 | -0.023 | 0.016 | -0.055 - 0.009 | .151 |  |
| ED-diagnosis ^b^ | 0.797 | 0.226 | 0.354 - 1.240 | **< .001** | 0.372 | 0.166 | 0.047 - 0.698 | **.025** | 0.480 | 0.170 | 0.146 - 0.814 | **.005** | 1.020 | 0.218 | 0.593 - 1.447 | **< .001** |  |
| BMI-SDS | 0.164 | 0.037 | 0.092 - 0.237 | **< .001** | 0.094 | 0.034 | 0.028 - 0.161 | **.006** | 0.129 | 0.033 | 0.065 - 0.194 | **< .001** | 0.290 | 0.042 | 0.207 – 0.372 | **< .001** |  |
| Random effects | variance | SE | 95% CI |  | variance | SE | 95% CI |  | variance | SE | 95% CI |  | variance | SE | 95% CI |  |  |
| Intercept | 0.839 | 0.048 | 0.750 - 0.939 |  | 0.783 | 0.038 | 0.711 - 0.862 |  | 0.730 | 0.043 | 0.651 - 0.818 |  | 1.213 | 0.055 | 1.110 - 1.327 |  |  |
| R^2^c | 0.451 | | | | 0.343 | | | | 0.360 | | | | 0.468 | | | |  |

*Note.* Abbreviations: ED, eating disorder; R^2^c, conditional R^2^-approximation; SE, standard errors (robust standard errors are displayed). Analyses are weighted with respect to age and sex. *p*-values < .05 are printed in bold. Experiential avoidance, mood, and negative thoughts are person-mean-centered.

^a^ male sex represents the base category.

^b^ absence of a 12-month eating disorder diagnosis represents the base category.

| **Table S7.** Associations between time-lagged disordered eating behaviors and subsequent experiential avoidance on the situational level (Model 3) | | | | | | | | | | | | | | | | |  |
| --- | --- | --- | --- | --- | --- | --- | --- | --- | --- | --- | --- | --- | --- | --- | --- | --- | --- |
|  |  |  |  |  |  |  |  |  |  |  |  |  |  |  |  |  |  |
|  | **Skipping eating**  **(predictor)** | | | | **Eating large amounts of food (predictor)** | | | | **Loss-of-control eating**  **(predictor)** | | | | **Restrained eating**  **(predictor)** | | | |  |
| Fixed effects | b | SE | 95% CI | *p* | b | SE | 95% CI | *p* | b | SE | 95% CI | *p* | b | SE | 95% CI | *p* |  |
| Intercept | 0.923 | 0.224 | 0.484 - 1.362 | **< .001** | 0.590 | 0.230 | 0.139 - 1.042 | **.010** | 0.566 | 0.234 | 0.108 - 1.024 | **.016** | 0.702 | 0.237 | 0.238 - 1.166 | **.003** |  |
| Time-lagged DEB | 0.008 | 0.008 | -0.008 – 0.023 | .331 | -0.001 | 0.009 | -0.019 - 0.018 | .942 | -0.012 | 0.006 | -0.023 - -0.001 | **.033** | 0.002 | 0.006 | -0.009 - 0.013 | .734 |  |
| Time difference | 0.001 | 0.001 | 0.000 - 0.003 | **.045** | 0.002 | 0.001 | 0.000 - 0.003 | **.044** | 0.002 | 0.001 | 0.000 - 0.003 | **.036** | 0.002 | 0.001 | 0.000 - 0.003 | **.048** |  |
| Time difference X Time-lagged DEB | -0.000 | 0.000 | -0.000 - 0.000 | .540 | 0.000 | 0.000 | -0.000 - 0.000 | .883 | 0.000 | 0.000 | 0.000 -0.000 | **.032** | -0.000 | 0.001 | -0.000 - 0.000 | .800 |  |
| Average DEB-value | 0.028 | 0.004 | 0.020 - 0.035 | **< .001** | 0.034 | 0.004 | 0.026 - 0.042 | **< .001** | 0.030 | 0.005 | 0.021 - 0.040 | **< .001** | 0.011 | 0.002 | 0.007 - 0.015 | **< .001** |  |
| Time-lagged mood | -0.002 | 0.001 | -0.004 - -0.001 | **.010** | -0.002 | 0.001 | -0.004 - -0.000 | **.034** | -0.002 | 0.001 | -0.003 - -0.000 | **.040** | -0.002 | 0.001 | -0.003 - -0.000 | **.037** |  |
| Time-lagged negative thoughts | 0.003 | 0.001 | 0.002 - 0.005 | **< .001** | 0.004 | 0.001 | 0.003 - 0.006 | **< .001** | 0.004 | 0.001 | 0.003 - 0.006 | **< .001** | 0.004 | 0.001 | 0.003 - 0.006 | **< .001** |  |
| Sex ^a^ | 0.143 | 0.057 | 0.032 - 0.254 | **.012** | 0.179 | 0.055 | 0.070 - 0.287 | **.001** | 0.156 | 0.056 | 0.045 - 0.266 | **.006** | 0.169 | 0.058 | 0.056 - 0.282 | **.003** |  |
| Age | 0.000 | 0.012 | -0.023 - 0.024 | .987 | 0.014 | 0.012 | -0.009 - 0.037 | .246 | 0.017 | 0.012 | -0.007 - 0.041 | .157 | 0.012 | 0.012 | -0.012 - 0.036 | .321 |  |
| ED-diagnosis ^b^ | 0.060 | 0.120 | -0.174 - 0.294 | .617 | 0.205 | 0.118 | -0.027 - 0.437 | .083 | 0.167 | 0.125 | -0.078 - 0.412 | .181 | 0.190 | 0.136 | -0.076 - 0.456 | .161 |  |
| BMI-SDS | -0.012 | 0.028 | -0.068 - 0.044 | .670 | 0.011 | 0.031 | -0.050 - 0.073 | .718 | 0.007 | 0.032 | -0.055 - 0.069 | .820 | -0.008 | 0.032 | -0.071 - 0.055 | .802 |  |
| Random effects | variance | SE | 95% CI |  | variance | SE | 95% CI |  | variance | SE | 95% CI |  | variance | SE | 95% CI |  |  |
| Intercept | 0.643 | 0.033 | 0.582 - 0.710 |  | 0.627 | 0.032 | 0.567 - 0.692 |  | 0.641 | 0.033 | 0.579 - 0.710 |  | 0.682 | 0.033 | 0.620 - 0.750 |  |  |
| R^2^c | 0.359 | | | | 0.364 | | | | 0.367 | | | | 0.366 | | | |  |

*Note.* Abbreviations: ED, eating disorder; DEB, disordered eating behavior; R^2^c, conditional R^2^-approximation; SE, standard errors (robust standard errors are displayed). Analyses are weighted with respect to age and sex. *p*-values < .05 are printed in bold. Each disordered eating behavior, mood, and negative thoughts are person-mean-centered.

^a^ male sex represents the base category.

^b^ absence of a 12-month eating disorder diagnosis represents the base category.

**References**

1. Kashdan TB, Goodman FR, Machell KA, et al (2014) A contextual approach to experiential avoidance and social anxiety: Evidence from an experimental interaction and daily interactions of people with social anxiety disorder. Emotion 14:769–781. https://doi.org/10.1037/a0035935

2. Heron KE, Scott SB, Sliwinski MJ, Smyth JM (2014) Eating behaviors and negative affect in college women’s everyday lives. Int J Eat Disord 47:853–859. https://doi.org/10.1002/eat.22292

3. Rao JNK, Scott AJ (1984) On chi-squared tests for multiway contingency tables with cell proportions estimated from survey data. The Annals of Statistics 12:46–60
